# Subchondral Bone Regeneration of the Porcine Knee Joint Using α-TCP Biocement Enriched with Manuka Honey—Qualitative Pilot Study

**DOI:** 10.3390/life16071192

**Published:** 2026-07-19

**Authors:** Filip Korim, Katarína Vdoviaková, Lenka Krešáková, Filip Humeník, Ján Danko, Zuzana Čriepoková, Jozef Bíreš, Kristína Čurgali, Zuzana Fagová, Marko Vrzgula, Mária Giretová, Ľubomír Medvecký, Radoslava Štulajterová, Roman Totkovič, Pavol Rusnák

**Affiliations:** 1 Department of Morphological Disciplines, University of Veterinary Medicine and Pharmacy in Košice, Komenského 73, 041 81 Košice, Slovakia; 2 Equine Clinic, University Veterinary Hospital, University of Veterinary Medicine and Pharmacy in Košice, Komenského 73, 041 81 Košice, Slovakia; 3 Ruminant Clinic, University Veterinary Hospital, University of Veterinary Medicine and Pharmacy in Košice, Komenského 73, 041 81 Košice, Slovakia; 4 Department of Histology and Embryology, Faculty of Medicine, Pavol Jozef Šafárik University in Košice, Šrobárova 2, 041 83 Košice, Slovakia; 5 Department of Anatomy, Faculty of Medicine, Pavol Jozef Šafárik University in Košice, Šrobárová 2, 041 83 Košice, Slovakia; 6 Division of Functional and Hybrid Materials, Institute of Materials Research of Slovak Academy of Sciences, Watsonova 47, 040 01 Košice, Slovakia; 7 AGEL Hospital a.s. Košice-Šaca, Lúčna 57, 040 15 Košice, Slovakia

**Keywords:** biocement, bone, calcium phosphate, manuka honey, pig, regeneration, subchondral bone

## Abstract

The treatment of osteochondral defects still poses a challenge, despite the fact that there are currently many methods and various materials designed for the regeneration of these tissues. The aim of our study was to evaluate the biocompatibility and bioactive potential of an innovative biocement enriched with manuka honey and α-tricalcium phosphate in a porcine model, with a focus on subchondral bone regeneration. Osteochondral defects of the medial femoral condyle were treated with the tested biocement in five animals, and five additional animals served as controls (untreated defect). After six months of observation, the regenerative potential of the therapeutic strategy was evaluated macroscopically, using imaging methods (X-ray, CT, and MRI) and histological examination. The studied biomaterial showed no cytotoxicity and proved to be biocompatible. In the in vivo system, based on imaging and histological methods, a typical trabecular organization with cellular elements has been observed in all animals treated with the tested biocement, indicating active remodeling and maturation processes. In animals with untreated defects, incomplete healing of the bone defect occurred, with limited areas of trabecular bone and incomplete boundaries between the cartilage and subchondral bone. Our pilot study, employing a multi-method approach to investigate the in vivo effects of a novel biocement on bone tissue regeneration, may highlight the potential contribution of biomaterials to the development of new therapeutic strategies in the context of osteochondral regeneration.

## 1. Introduction

Subchondral bone (SB) forms a transition between articular cartilage and cancellous bony tissue; this structure consists of trabecular bone tissue with fatty bone marrow and a rich presence of pores of various sizes [1]. The thickness of SB varies in the regional position from 10 µm to 3 mm [2,3]. Like bone tissue, SB is a biphasic material predominantly composed of hydroxyapatite crystals, giving it strength. The organic substances are dominated by collagen I, proteoglycans, and glycosaminoglycans, which are responsible for their elasticity [4]. Compared to long cortical bones, SB has a 10× greater ability to respond to stimuli (stress, forces), resulting in its physical adaptation to these stimuli in the joint. The tissue is supplied by the highly dense vasculature and is well innervated, and its role is to provide mechanical and nutritional support for the articular cartilage [3]. This results in a complex and extensive local response to physiological stimuli, but also stress stimuli in the joint, which leads to internal changes within the SB [2]. Subchondral bone is involved in the formation of the osteochondral unit, which is crucial for understanding osteochondral injury mechanisms [2,5].

Cartilage tissue defects often not only affect the cartilage tissue itself but also the SB, resulting in the development of osteoarthritis of the affected joint [6,7]. One of the phenomena accompanying osteoarthritis is the presence of osteophytes in the affected joint. Behind the development of osteophytes, which are responsible for joint degeneration, there are various changes in the structure of trabecular bone [8,9]. Subchondral bone shows osteoporotic changes in early and advanced stages of osteoarthritis, increasing its porosity and decreasing the volume and complexity of bone trabeculae. The cause of the change in the volume and complexity of trabecular bone is its microdamage due to the changes in the load on the affected joint. In addition to these changes, subchondral sclerosis is described in the literature, which occurs after the initial reduction in bone mass. In later stages, significant abnormalities of the bone and its cysts appear [10]. Damage to the SB not only leads to the development of osteoarthritis but also to other diseases associated with damage to articular cartilage, namely osteochondrosis, osteochondral fragmentation (chip fractures), and condylar fractures [2].

Regeneration of cartilage, but also of the SB itself in osteochondral defects/injuries, is a great challenge. Currently, well-known and used techniques include bone marrow stimulation, microfractures, subchondral drilling, and abrasion arthroplasty [11,12]. Nowadays, bioengineering and regenerative medicine techniques are coming to the forefront with the aim of creating an ideal “native” tissue with the help of various transplants (autografts, allografts, xenografts), scaffolds, and biocements, which can be enriched with various cellular components and biologically active molecules [13,14].

Calcium phosphates (CPs) are the most represented inorganic components within bone tissue. Osteoblasts are responsible for the release of Ca^2+^ and PO_4_^−^ ions into the collagen matrix and the formation of substance–hydroxyapatite Ca_5_(PO_4_)_3_(OH) [15,16]. Calcium phosphates are frequently used biomaterials in the regenerative medicine field due to their similarity to natural hydroxyapatite in bone since the 1980s [16,17]. CPs have excellent osteoconductive and osteoinductive properties and the ability to osteogenically stimulate bone marrow mesenchymal stem cells [18,19,20]. The angiogenetic potential of CP biomaterials is crucial for bone neoformation [21]. The biocompatibility of CP materials is conditioned by their solubility in body fluids, their ability to release and increase the concentration of Ca^2+^ and PO_4_^−^ ions at the site of implantation, as well as influence the adhesion and proliferation of osteoblasts and thus the synthesis of newly formed bone. They are also proven to influence the expression of osteoblastic differentiation markers such as type I collagen, alkaline phosphatase, bone matrix proteins, osteopontin, osteocalcin, bone sialoprotein, osteonectin, and Runt-related transcription factor 2 (RunX2). The release of Ca^2+^ and P^−^ ions is considered to be the most crucial, affecting osteogenic cells, tissues, metabolic and activation processes and pathways [22,23].

The domestic pig is frequently used as a biomodel for testing the regenerative potential of a wide spectrum of biomaterials, such as CPs. Some factors, such as close similarity of bone’s anatomy (macroscopic and microscopic), bone mineral composition, bone density, and speed of bone remodeling (1.2–1.5 µm per day in pig vs. 1.0–1.5 µm per day in human), make pigs a very suitable animal model for applied research to human medicine [24,25]. There are numerous studies that use domestic pigs as models for osteochondral and bone trauma regeneration by using CPs. Domestic pigs have been used as useful animal models for the study of craniofacial defects, osteochondral defects, and long bone (femur, tibia, radius) defect treatments [26,27,28,29].

Honey has been known for a long time to treat skin wounds, but there is little knowledge about the possibility of utilizing CP biocements/honey composites for the treatment of bone tissue defects, and to consider honey as a possible stimulating component. The honey in relation to bone can protect the bone tissue against oxidative stress caused by reactive oxygen species; it has an anti-inflammatory effect primarily due to the presence of polyphenols, as well as antibacterial properties, which arise from glyoxal (GO) or methylglyoxal (MGO) [30]. It was revealed that oxidative stress negatively affected bone mineral density, reduced bone formation, and induced osteoporosis [31], and a high production of reactive oxygen species (ROS) inhibited osteoblast differentiation and promoted apoptosis [32].

The hypothesis of our research was that the application of an innovative biomaterial based on calcium phosphate with the addition of manuka honey (MH) is safe and contributes to the regeneration of subchondral bone in a porcine model. The aim of our study was to investigate the therapeutic potential of α-TCP+M25 biocement in artificially created subchondral bone defects of the left medial femoral condyle in a porcine animal model. The regenerative potential was assessed based on computed tomography, magnetic resonance imaging, histopathological examination, and immunohistochemical examination.

## 2. Materials and Methods

### 2.1. Cement Preparation

The α-tricalcium phosphate (α-TCP) powder was synthesized using a solid-state reaction from a mixture of calcium carbonate (CaCO_3_, analytical grade, Sigma-Aldrich, Saint Louis, MO, USA) and calcium hydrogen phosphate anhydrous (monetite, DCPA; CaHPO_4_, Ph.Eur., Fluka, Steinheim, Germany). The mixture was heated at 1320 °C for 2 h to obtain the α-TCP phase. Subsequently, the resulting α-TCP was dry-milled in a planetary ball mill (Retsch PM100) at 350 rpm using an agate milling vessel and agate balls for 2 h, and the final particle size d50 = 8.9 μm (Mastersizer 2000 E, Malvern Instruments Ltd., Malvern, Worcestershire, UK) [33].

Manuka honey (M25) (Activion^®^, medical grade, Advancis Medical, Nottingham, UK) was dissolved in a 1% NaH_2_PO_4_ solution (analytical grade, Sigma-Aldrich, Steinheim, Germany), which served as the liquid component of the biocement. The liquid phase contained 5% (*w*/*v*) honey. The cement paste (α-TCP+M25) was prepared by mixing the α-TCP powder with the liquid phase at a powder-to-liquid ratio (P/L) of 2.6, which was optimal from the point of view of viscosity and had good manipulation with the paste. The composite cement contained about 2 wt% of honey.

### 2.2. Characterization of Microstructure, Setting Time, and Mechanical Properties

The compressive strength (CS) of the cements was determined using a universal testing machine (LR5K Plus, Lloyd Instruments Ltd., West Sussex, UK) equipped with a 5 kN load cell. The measurements were performed at a crosshead speed of 1 mm/min, and the results are reported as mean ± standard deviation (n = 4). For testing, cement pastes were packed into stainless steel cylindrical molds (6 mm in diameter and 12 mm in height), allowed to set at 37 °C under 100% humidity for 10 min, and subsequently immersed in simulated body fluid (SBF) at 37 °C for 1 week (500 mg sample and 40 mL SBF), which was sufficient time for stabilization of micro-structure with the high degree of α-TCP transformation. Note that for in vivo testing, the long-term in vitro analysis of α-TCP transformation in SBF has only limited significance due to the strong effect of cells, which interact with CPs and resorb and remodel material with the formation of new bone tissue. The simulated body fluid was prepared according to [34].

The phase composition of the samples was analyzed by X-ray diffraction (XRD) using a Philips X’PertPro diffractometer (Malvern Panalytical B.V., Eindhoven, the Netherlands) with Cu Kα radiation at 50 mA in the 2θ range of 20–40°. The semi-quantitative content of TCP phases was calculated from the comparison of the integrated intensities of TCP line (034 plane) after line fitting using the Profit XRD program according to equation [35]:

R_TCP_ = 100 × I_TCP_/(I_TCP_ + I_HAP_) (1)

where I_TCP_ corresponds to the intensity of reflection from the α-TCP (034)plane, and I_HAP_ corresponds to the intensity of reflection from the HAP (002)plane.

The microstructure of the fractured cement surfaces was examined using field emission scanning electron microscopy (FE-SEM, JEOL JSM-7000F, JEOL Ltd., Tokyo, Japan) after carbon coating of the samples. The final setting time of the cement pastes was determined using the Gilmore needle method [36].

### 2.3. In Vitro Cytotoxicity Testing of the Cement Pastes

The biological evaluation of the prepared cements was performed in accordance with the general principles of ISO 10993-5, utilizing both direct contact and extraction methods [37]. Prior to the in vitro experiments, the cement samples were sterilized by UV irradiation for 30 min on each side.

**Direct Contact Assay:** The direct contact cytotoxicity and cell proliferation on α-TCP and α-TCP+M25 cements were assessed using MC3T3-E1 Subclone 4 cells-CRL-2593™ (ATCC, Manassas, VA, USA). Sterilized cement disks (6 mm in diameter and 1 mm thick) were placed into 48-well culture plates (TPP Techno Plastic Products AG, Trasadingen, Switzerland). Subsequently, 5 × 10^4^ cells suspended in 1 mL of culture medium (Eagle’s Minimum Essential medium—EMEM supplemented with 10% fetal bovine serum (FBS) and 1% antibiotic–antimycotic solution; all from Sigma-Aldrich) were seeded directly onto the sample surfaces (400 µL/2 × 10^4^ cells/well). All experiments were conducted in triplicate. Cells cultured in complete culture medium without any sample served as the negative control (NC). The culture medium was replaced three times per week.

Cell proliferation and morphology were evaluated after 2, 8, and 15 days of cultivation using live/dead fluorescent staining (fluorescein diacetate (FDA) and propidium iodide (PI)). The samples were observed using an inverted fluorescence microscope - Leica DM IL LED (Leica Microsystems GmbH, Wetzlar, Germany) equipped with a blue filter. To prevent the loss of weakly adherent dead cells, the cell culture medium was carefully aspirated, and the staining solution was applied directly to the cell monolayer without prior PBS washing. In this assay, intracellular enzymes in viable cells convert FDA into a green fluorescent compound, while PI penetrates cells with compromised membranes and stains non-viable cells red. The average population density of live cells after 48 h, 8 d, and 15 d of cultivation on the cement surfaces (n = 3) was calculated from three images (at magnification 10×) and expressed as the number of live cells per 1 mm^2^ (mean ± standard deviation (SD)). Statistical significance (*p* < 0.05) was evaluated using one-way ANOVA (Statmost for Windows).

**Cytotoxicity of extracts:** Cytotoxicity of cement extracts (α-TCP and α-TCP+M25) was evaluated as follows: Cement samples were incubated in culture medium (EMEM) supplemented with FBS at a concentration of 0.2 g/mL for 24 h at 37 °C to obtain the extracts. Preosteoblast MC3T3-E1 cells (10^4^ cells in 100 µL of culture medium/well (105/mL) were seeded into wells of 96-well plates (SARSTEDT AG & Co. KG, Nümbrecht, Germany). After 24 h of cultivation, the culture medium was removed and replaced with the prepared extracts. Following another 24 h incubation with the extracts, the medium was replaced with fresh culture medium. Cells cultured in extract-free culture medium served as a negative control (NC). Cell viability was then quantitatively determined using the MTS-based (3-(4,5-dimethylthiazol-2-yl)-5-(3-carboxymethoxyphenyl)-2-(4-sulfophenyl)-2H-tetrazolium) proliferation assay (CellTiter 96^®^ Aqueous One Solution Cell Proliferation Assay, Promega, Madison, WI, USA). The yellow tetrazolium salt (MTS) is reduced by viable cells to a water-soluble formazan dye. This product was quantified directly in the cell culture medium by measuring absorbance at 490 nm with a UV-VIS spectrophotometer (Shimadzu, Kyoto, Japan).

#### Statistical Analysis

A one-tailed ANOVA statistical analysis (*p* < 0.05) was performed using the Statmost for Windows program. The relative viability of osteoblasts was calculated as the ratio of the average formazan absorbance in the samples (n = 3) to the average formazan absorbance in the negative control. The results are presented as mean ± SD. The cytotoxicity of the samples was statistically tested in relation to the cytotoxicity threshold represented by 70% of the viability of the negative control. In this way, the hypothesis was tested whether the average relative viability of the samples was higher than 70% of the viability of the negative control.

### 2.4. Experimental Creation of the Osteochondral Defect

A total of 10 healthy pigs—Slovak White Pig Breed of both sexes, weighted 95.4 ± 3.62 kg—were included in our study. The average age of the pigs was 6.9 ± 0.87 months. All animals were clinically examined before experimental surgery, especially for orthopedic issues such as lameness, claw diseases, and developmental disorders. Pigs were housed in separate pens at the Swine Clinic of the university, and they had ad libitum access to feed and water before and after surgery.

The animals were divided into two groups of 5 individuals. In one group, α-TCP+M25 biocement was applied to the artificially created osteochondral defect of the medial femoral condyle. In the second group of animals, these defects were allowed to heal spontaneously. The right limb´s medial femoral condyle was left intact and served as a comparison with native cartilage.

Before the surgery itself, the pigs were intramuscularly sedated in the pens with a mixture of drugs (MIX), which consisted of 50 mg/mL tiletamine and 50 mg/mL zolazepam (ZOLETIL 100 Vet., Virbac, Nice, France), 2.5 mL ketamine (Ketamidor 100 mg/mL, VetViva Richter GmbH, Wels, Austria) and 2.5 mL of xylazine (Xylased 100 mg/mL, Bioveta SK spol., s.r.o., Nitra, Slovakia). The dose for successful preoperative sedation of the animal was 4 mL/100 kg MIX + 1.5 mL/100 kg butorphanol (Butomidor 10 mg/mL, VetViva Richter GmbH, Wels, Austria). To maintain anesthesia, we used 0.5 mL–1.0 mL of a pre-prepared MIX of anesthetics administered intravenously.

We accessed the left medial condyle of the femur under strict aseptic conditions through a medial skin incision and dissection of soft tissues (fascia and muscles). After dissecting the joint capsule and visualizing the left medial condyle itself, we created a 10 × 10 mm (diameter × depth) circular defect using a surgical drill. Subsequently, the defect was filled with pre-prepared α-TCP+M25 biocement paste, which we obtained by mixing the powder phase with the liquid phase (Figure 1). After application of the material, the surgical wound was closed in all anatomical layers using absorbable suture material, and we performed post-operative X-ray examination (Figure 2).

Post-operatively, antibiotics were applied to the animals—oxytetracycline dihydrate in a dose of 1 mL/10 kg (Alamycin LA a.u.v., Norbrook, Newry, UK)—every other day for one week, and non-steroidal anti-inflammatory drugs—flunixin meglumine in a dose of 2 mL/45 kg (Flunixin a.u.v., Norbrook, Newry, UK)—were administered every day for one week. During the post-operative period, we monitored the mobility of the pelvic limb, the degree of lameness according to Grégoire et al. [38], the presence of inflammation, and other orthopedic problems.

After the observation period (six months), the experimental animals were humanely euthanized by intravenous administration of 90 mg/kg thiopental (Thiopental VUAB 1.0 g, VUAB Pharma a.s., Roztoky u Prahy, Czech Republic). Before the administration of thiopental, the animals were under deep sedation, which was induced by the intramuscular application of an anesthetic mixture of drugs. Immediately after euthanasia, the left pelvic limbs of the animals were ex-articulated in the hip joint for the purpose of macroscopic examination, X-ray examination, computed tomography (CT) examination, magnetic resonance imaging (MRI) examination, and histological examination.

### 2.5. Evaluation of the Therapeutic Effect of Biocement

#### 2.5.1. Macroscopic Evaluation

Osteochondral defects were evaluated after dissection of the muscles and joint capsule of the left pelvic limb. In addition to the cartilage, we observed the surrounding synovial structures of the joint (joint capsule, synovial membrane, synovium) as well. Macroscopically, we assessed the thickness of the joint capsule and synovial membrane, the joint effusion, the presence of synovial fluid, its density and viscosity, and color.

At the site of the defect, we evaluated its appearance, color, regularity, tissue structure, biocement remains, and its integration with the surrounding cartilage. Overall, we also focused on signs of inflammation in the knee joint area (edema, hyperemia) and the presence of osteophytes. These properties were evaluated in both groups of animals and compared with the contralateral native cartilage.

#### 2.5.2. Magnetic Resonance Imaging and Radiological Evaluation

The regenerative response of the articular cartilage and subchondral bone in the left porcine knee joints was evaluated by post-mortem imaging examinations performed six months after the surgical procedure. The assessment included X-ray, CT, and MRI.

Radiographic evaluation was conducted using lateral projections of the knee joint obtained with a digital X-ray system (Philips Digital Diagnost, Delft, the Netherlands).

Computed tomography examinations of the knee were performed using a computed tomography scanner (Philips Brilliance 40-slice CT, Philips Medical Systems Nederland B.V., Best, the Netherlands) in the axial plane. These images were used to evaluate the newly formed tissue in the knee joint region.

Magnetic resonance imaging was performed in one plane using a dedicated Zenith solenoid knee coil to ensure optimal visualization of the articular cartilage. The axial plane was acquired using a T2-weighted sequence (T2 AX) with a repetition time (TR) of 4500 ms and an echo time (TE) of 84 ms.

All images were acquired with ultra-high spatial resolution and a slice thickness of 3.5 mm using a 1.2 T open MRI system (Hitachi Oasis, Hitachi Medical Systems Holding AG, Tokyo, Japan). The MRI and CT images were subsequently transferred to the TOMO CON PACS network and analyzed using specialized Tatra Med image analysis software https://tatramed.sk/en/tomocon-pacs/ accessed on 16 July 2026.

#### 2.5.3. Histological Examination

Samples from the newly formed tissue sites were harvested using the OATS kit (Arthrex, Naples, FL, USA). These cylinders were then divided into 5 representative symmetrical areas and were fixed in 10% neutral buffered formalin at room temperature. After fixation, decalcification was carried out using a 25% EDTA solution (CentralChem, Bratislava, Slovakia). Completion of decalcification was assessed mechanically using a needle or forceps. Subsequently, the specimens were dehydrated through a graded ethanol series, cleared in xylene, and embedded in paraffin. Tissue sections were examined under high magnification (×400). Images were captured using an Olympus BX50 (Olympus, Tokyo, Japan) light microscope equipped with an Olympus SP350 (Olympus, Tokyo, Japan) digital camera.

##### Hematoxylin–Eosin (H&E) Staining

Hematoxylin–eosin staining is a standard technique widely used for the routine evaluation of tissue architecture. This staining combination enables clear differentiation of cellular components and extracellular structures.

Paraffin sections were first deparaffinized in xylene and rehydrated through decreasing concentrations of ethanol. The sections were then stained with hematoxylin for 5 min, rinsed, and counterstained with eosin for 3 min. After staining, the slides were dehydrated in graded alcohols, cleared in xylene, and mounted using a synthetic resin medium (Entellan; Merck, Darmstadt, Germany). All stained sections were further analyzed for histopathological features and morphometric parameters.

##### Safranin-O/Fast Green Staining

Safranin-O staining (ranging from red to orange) indicates the presence of a sulfated glycosaminoglycan-rich matrix, typical of hyaline cartilage and chondroid tissues. In contrast, Fast Green serves as a counterstain for collagenous extracellular matrix and bone, producing a green to blue-green coloration depending on staining conditions.

Sections were deparaffinized in xylene and rehydrated through graded ethanol solutions. Nuclear staining was performed using Weigert’s iron hematoxylin for 5 min, followed by rinsing in running water. The sections were then briefly treated with acid alcohol and washed three times in distilled water.

Next, sections were stained with 0.02% Fast Green for approximately 1.5 min and briefly rinsed in 1% acetic acid (30 s) to remove excess dye and enhance contrast. Subsequently, slides were stained with 0.1% Safranin O for 15 min, rapidly dehydrated through graded ethanol, cleared in xylene, and mounted with a coverslip.

##### Immunohistochemical Analysis

Decalcified tissue sections were deparaffinized and rehydrated prior to staining. Endogenous peroxidase activity was blocked using 3% hydrogen peroxide in methanol. Antigen retrieval was performed before primary antibody incubation using a PT-Link system (Dako, Glostrup, Denmark) with a high pH retrieval buffer.

For immunohistochemical detection, sections were incubated with primary antibodies. Detection was carried out using a two-step indirect immunoperoxidase method. Both primary and secondary antibodies were applied at optimized dilutions (Table 1). Positive immunoreactivity was visualized using diaminobenzidine (DAB; Sigma-Aldrich, Saint-Louis, MO, USA). Cell nuclei were counterstained with Mayer’s hematoxylin, and slides were mounted using the Pertex mounting medium. To verify staining specificity, negative controls were included by omitting the primary antibody. This control procedure was applied across all experimental groups.

## 3. Results

### 3.1. Cement Properties

#### 3.1.1. XRD Analysis of Cement

X-ray diffraction analysis of the initial powdered cement phase confirmed that α-TCP (JCPDS 29-0359) was the dominant crystalline phase, accompanied by approximately 9% of β-TCP as a secondary phase (JCPDS 09-0169) (Figure 3). Comparison of the diffraction patterns of the composite biocement α-TCP+M25 after 7 days of immersion in SBF under physiological conditions indicated that more than 80% of the α-TCP phase was transformed into nanocrystalline hydroxyapatite (JCPDS 72-1243). The hydroxyapatite crystallinity sizes calculated from the (002) hydroxyapatite plane using the Scherrer equation were 31 nm. Residual peaks corresponding to the β-TCP phase were still detectable in the diffraction patterns.

#### 3.1.2. Characterization of Biocement Microstructure, Setting Time, and Compressive Strength

The microstructure of the α-TCP biocement revealed a mixture of irregularly shaped particles and granular agglomerates of hydroxyapatite (HAP). The presence of these agglomerates contributed to the formation of spherical and irregular macropores with sizes up to 10 μm, which likely resulted from the detachment of weakly bonded HAP nanoparticle clusters from the cement matrix (Figure 4a). In addition, the agglomerates consisted of rod- and plate-like HAP nanoparticles with lengths up to 1 μm (Figure 4b). These structures represented the predominant morphological features and were interconnected with very fine nanocrystalline, granular HAP particles of submicrometric size within the cement matrix.

The incorporation of MH in the α-TCP+M25 samples promoted the development of a denser microstructure characterized by a higher proportion of micropores of approximately 1 μm located between agglomerates of a very fine hydroxyapatite matrix (Figure 4c). Furthermore, compact irregular agglomerates with sizes up to 5 μm were observed in the fractured surfaces (Figure 4d), which represent unreacted remains of the original α-TCP cement particles tightly coated by the cement matrix. These structures may correspond to remnants of the original α-TCP particles surrounded by newly formed fine HAP crystals.

The final setting time of the α-TCP+M25 cement was nearly doubled compared to the α-TCP cement, reaching 28 min. In addition, the compressive strength of α-TCP (39 ± 2 MPa) was significantly higher than that of the α-TCP+M25 sample (27 ± 3 MPa).

In addition, the compressive strength of α-TCP (39 ± 2 MPa) was statistically significantly different (*p* < 0.005, one-way ANOVA, n = 3) from that of the α-TCP+M25 sample (27 ± 3 MPa).

#### 3.1.3. Cytotoxicity Analysis of Cement Extracts and Osteoblast Proliferation on Cement Surfaces

The standard MTS assay revealed no cytotoxic effects associated with the cement extracts. The viability of osteoblasts cultured in extracts from α-TCP and α-TCP+M25 cements reached 108 ± 11% and 100 ± 10% relative to the negative control, respectively. Both values significantly exceeded (*p* < 0.05) the commonly accepted cytotoxicity threshold of 70% for extract-based assays (Figure 5).

In addition, live/dead fluorescence staining confirmed the biocompatibility of the cements after 2 days (Figure 6a,b), 8 days (Figure 6c,d), and 15 days of cultivation (Figure 6e,f). The cells demonstrated good adhesion, spreading, and high cell density on all tested samples. Importantly, no non-viable cells were observed on the cement surfaces, regardless of the cement composition. Over time, cell density progressively increased, with the α-TCP+M25 cement exhibiting a more confluent cell layer compared to α-TCP (without honey) after 8 and 15 days of culture.

The average population density of live proliferating cells on the cement substrate surfaces was calculated from three images taken from different areas of the substrate (Figure 7). It was found that the average population densities on the α-TCP+M25 samples were higher (statistically significantly different) than those on the α-TCP sample without the addition of honey at selected times. Statistical analysis of the data in Figure 7 clearly revealed a time-dependent increase (statistically significant difference) in average cell density on the surfaces of both groups. By day 15, the mean cell density on the M25-modified surface reached approximately 1500 cells/mm^2^, nearly 1.6 times higher than that of the α-TCP group.

### 3.2. Post-Operative Recovery of Animals

We did not notice any problems during the surgery itself (waking up from anesthesia, significant bleeding, hypothermia). The animals woke up spontaneously in the pens without any problems. Ten hours after the surgery, the animals were taking food and water normally and were interested in the surroundings.

We noticed slight lameness in all animals approximately 1 week after the surgery. In two animals, lameness persisted until the third week after the surgery (group without filling the defect with biocement). The animals were able to fully weigh the operated limb in the second week after biocement implantation. The surgical wound showed mild hyperemia and edema in the first week after the surgery. However, wound abscessation and wound dehiscence were not observed in any animal.

### 3.3. Macroscopic Evaluation

The knee joints of the experimental animals were carefully opened and examined immediately after euthanasia. We did not record any periarticular and intra-articular pathological processes in any of the animals 6 months after the operation. The joint capsule had a normal appearance and consistency, and the synovial membrane did not show signs of synovitis. The synovial fluid was in an adequate amount without alterations in color and viscosity. There was no fibrin in the joint cavity, and no adhesions between the synovial membrane and neocartilage were observed.

In the group of animals in which biocement was implanted into the defect, we visually detected neocartilage, which was almost identical to the surrounding intact cartilage. In the center of 2/5 animals, there was a scar-like structure, which differed from the native cartilage by its lower location (Figure 8A). However, the neocartilage was smooth, moist, and without prominences, and was very similar to, if not identical to, intact healthy cartilage (Figure 8C).

In spontaneously healed osteochondral defects, we did not observe nearly identical cartilage tissue, but rather newly formed cartilage that overlaid the native cartilage and differed mainly in its color and rougher surface. The new cartilage consisted of prominences and depressions that formed transition zones between healthy, intact cartilage (Figure 8B). The properties of this regenerate did not correspond to healthy intact cartilage to any extent (Figure 8C).

### 3.4. MRI and Radiologic Evaluation

In the group of animals treated with manuka honey-containing biocement, lateral radiographs showed a well-preserved and uniformly distributed joint space without signs of osteophyte formation (Figure 9A) or deformities of the femur, femoral condyles, or tibia. Conventional radiography also enabled the assessment of structural changes in the adjacent SB, such as subchondral osteosclerosis or cyst-like lesions. In this experimental group, no pathological changes indicative of progressive joint degeneration were detected. Overall, the radiographic findings in the biocement-treated group revealed no abnormal alterations.

Similarly, radiographic evaluation of the animals with spontaneous healing demonstrated a uniform joint space between the epiphyses and no pathological changes in the surrounding soft tissues (Figure 9B). However, conventional radiography indicated only partial healing of the osteochondral defect in this group (Figure 9B).

CT images acquired in the axial plane in the group of animals treated with the α-TCP+M25 biocement demonstrated reconstruction of the tissue in the defect area. The original knee joint defects were filled with newly formed bone tissue, with an average cortical bone density of 1620 HU and a trabecular bone density of approximately 620 HU. The measured values were comparable to the density of the surrounding healthy tissue. In the group of animals with spontaneous healing, incomplete defect filling was observed, along with lower bone density values.

Evaluation of the defects in the axial plane (Figure 10) revealed complete integration of the newly formed tissue without visible alterations in the structure or integrity of the examined tissues in animals treated with the biocement, compared with the group in which the defect was left to heal spontaneously (Figure 11).

The results of the CT examination confirmed the conclusions obtained from morphological and radiographic analyses. The treated defects showed a smooth surface of regenerated cartilage with a homogeneous internal structure and complete integration with the surrounding native articular tissue, as observed on MRI images. Cross-sectional evaluation of the osteochondral defect further confirmed complete restoration of the osteochondral unit, with the regenerated cartilage reaching a thickness of approximately 1.5 mm.

In all animals treated with biocement, the signal intensity measured within the repair tissue was comparable to that of the adjacent healthy cartilage layers and the underlying SB.

Comprehensive MRI analysis following implantation of the α-TCP+M25 biocement demonstrated complete filling of the defect. The regenerated SB displayed homogeneous morphology.

Furthermore, MRI findings indicated that after the induction of an osteochondral defect on the medial femoral condyle and implantation of the α-TCP+M25 biocement, sclerotic changes developed around the defect margins, suggesting an active healing response. Regeneration of cartilage tissue was evident at the defect site, and the thickness, structural organization, and signal characteristics of the repaired tissue were comparable to those of native healthy cartilage.

These observations indicate that the healing process in knees treated with α-TCP+M25 biocement was enhanced six months after implantation compared with the group undergoing spontaneous healing. The signal properties of the regenerated tissue were similar to those observed in control knees (Figure 12A,B).

In contrast, animals in the spontaneous healing group exhibited an irregular articular surface with shallow superficial ulcerations and morphological abnormalities such as fraying, fissuring, and fibrillation, although without exposure to the SB. MRI assessment in this group demonstrated incomplete repair six months after surgery, with newly formed tissue occupying approximately 50% of the initial defect volume (Figure 13). Additional tissue evaluation revealed reduced signal intensity in the central region of the defect, indicating insufficient structural regeneration.

### 3.5. Histological Evaluation

In a healthy intact medial condyle of the right femur, the SB shows a well-developed network of trabecular bone with healthy bone architecture. The trabeculae are composed predominantly of lamellar bone with focal areas of woven bone, reflecting physiological bone remodeling. Intertrabecular spaces are filled with well-vascularized bone marrow, with physiological cellular composition (Figure 14A).

On the surface of the bone trabeculae population, osteoblasts are present, while osteocytes are completely embedded within the bone matrix (Figure 14B). The overlying articular cartilage is intact, with preserved morphology and a well-defined osteochondral interface.

The histomorphological findings are consistent with physiological SB structure and homeostatic remodeling without evidence of pathological changes.

In a group of animals where osteochondral defects were filled with α-TCP+M25 biocement, the SB is characterized by extensive formation of new bone trabeculae composed mostly of woven bone with focal areas of more mature lamellar bone. The trabeculae form a dense and interconnected network with intertrabecular spaces filled with forming bone marrow, and blood vessels present (Figure 15A).

The surfaces of the trabeculae are lined by osteoblasts, and within the trabeculae, the osteocytes are embedded in the bone matrix, reflecting ongoing bone formation and maturation (Figure 15B). At the osteochondral interface, regions of newly formed cartilage with chondrocytes embedded in the extracellular cartilage matrix are present adjacent to the bone compartment.

Intensive strong positivity of the osteocalcin and osteopontin in osteoblasts confirmed an active repair and remodeling process in defects filled with biocement. On the other hand, in osteocytes, we observed a sporadic positivity of both markers (Figure 16A,B).

Histomorphological findings demonstrate active SB remodeling with trabecular formation and maturation, consistent with a reparative response following defect creation after the application of biomaterial.

In the group of animals where osteochondral defects were left to heal spontaneously, in the SB, we observed irregularly organized and rather disrupted trabecular bone, along with predominant formation of woven bone and limited areas of more mature lamellar bone, reflecting ongoing bone regeneration. The trabecular architecture appears discontinuous and less organized compared to the physiological bone. There is no clear border between the cartilage, where the defect area is predominantly filled with a well-developed fibrotic callus, which extends into the subchondral region (Figure 17A). Intertrabecular spaces are partially filled with vascularized connective tissue, with numerous blood vessels, indicating active repair and granulation-like tissue formation rather than a fully restored bone marrow architecture (Figure 17B).

Histomorphological findings correspond with incomplete SB regeneration during spontaneous healing, characterized by persistent fibrous tissue, immature woven bone formation, and ongoing remodeling without a full restoration of the normal trabecular structure of the SB. There is no clearly defined boundary between cartilage and SB, suggesting incomplete structural restoration of the osteochondral unit.

## 4. Discussion

Calcium phosphate cements based on α-TCP are widely used in dentistry, implantology, maxillofacial surgery, and orthopedic reconstructive surgery [33]. According to dos Santos et al. [39], these cements provide several advantages, such as the possibility of preparing the final cement paste during surgery, excellent contact between bone and cement, and, most importantly, the material is biocompatible and bioactive, which allows its wide applications. Among other advantages, these cements can serve as carriers for various substances, including ions, drugs, bioactive compounds, and cells [27,40]. Enrichment of CPs cement with various inorganic and organic components has often modified and increased their regenerative potential in in vitro and in vivo studies [28,41,42,43,44,45,46].

From the point of view of microstructures, the difference between α-TCP cement and honey composites was in the number of rod- or plate-like HAP particles created during the hydrolysis of the α-TCP phase and the refinement particles in the composite, as well as a lower fraction of larger pores, about 10 μm, and a higher number of approx. 1 μm pores found in the compositecements. It is worth noting that the calcium-deficient HAP particles with plate-like morphology are formed during α-TCP transformation in water at 37 °C [47]. It is also worth noting that the total porosities of cement were close to 50% and were not statistically significantly different.

It is well known that sugars like monosaccharides strongly suppressed the growth of HAP particles in supersaturated solutions of CPs [48], and it was found that the addition of glucose to freshly precipitated HAP particles at neutral pH conditions (similar to these in M10 composite) caused the strong interaction between glucose with OH groups in HAP [49] and the formation of the stable surface complexes between Ca^2+^ or phosphate ions with glucose, which support the refinement of HAP particles in the cement. Similar effects on the reduction in growth of HAP particles have been observed with flavonoids or other polyphenolic acids (found in honey) [50,51]. Thus, there is no doubt that the above-discussed components of honey were the reason for the inhibition of both the nucleation and growth of HAP particles in composite cement.

It is clear that a strong adsorption of the honey components on the surface of CP particles hinders the diffusion of ions during the hydrolysis of α-TCP and is a reason for the prolongation of the setting times of M10 cement. In addition, the adsorbed molecules of honey components changed the surface charge, especially in the case of polyphenols, which can cause repulsion of particles and the reduction in mutual bonding in composite cement with a decrease in CS. Similar effects were identified in published papers [52,53].

In the study by Oh et al. [54], α-TCP alone and with the addition of chitosan were tested in vitro for their effect on the adhesion, proliferation, and cytotoxicity of MC3T3-E1 cells. It was found that α-TCP supported cell viability, which was significantly higher (*p* < 0.05), similar to our case. The authors also examined osteoclastic activity using TRAP staining and the presence of multinucleated cells (over three nuclei). This activity was also significantly higher in cement, based on our results, but also based on the results of Oh et al. [54], where it can be concluded that α-TCP is a very suitable carrier used in regenerative medicine in terms of the effect on cell viability. In bone tissue regeneration, the activity of osteoblasts and osteoclasts has its place and is necessary to ensure a correct and smooth regeneration process. It was also shown that chitosan stimulated this activity, and its addition to tricalcium phosphate seems promising [54,55].

In addition to CP cements, which are enriched with natural additives, another very interesting and promising solution for the treatment of bone defects is the self-fitting 4D-printed bone tissue scaffold [56,57]. In the Choudhury et al. [56] study, polylactide-co-trimethylene carbonate (PLMC) was used, which was nanoengineered with polydopamine nanoparticles (PDA), where they demonstrated excellent osteogenic potential and increased alkaline phosphatase activity in vitro. They also showed very good properties and bone regeneration in the area of the tibia, mandible, and teeth in the therapy of critical bone defects in rabbits [56]. These 4D nanocomposites allow for image-guided placement through minimally invasive techniques, which facilitate precise implantation and postoperative monitoring [57].

One of the complications in implant surgery is the inflammatory process, which is caused by both Gram-negative and Gram-positive bacteria. Nowadays, the application of systemic antibiotics is very common, and multidrug resistance is associated with it. As mentioned, CP cements can serve as carriers of various substances with antimicrobial properties, such as ions (Ag^+^, Cu^2+^, Zn^2+^), but also honey [19,46,58,59,60,61].

Honey is a biological and bioactive compound that contains a high concentration of sugars (glucose, fructose), polyphenols, minerals, and vitamins. The varying concentrations and proportions of these components are responsible for the antioxidant, anti-inflammatory, antibacterial, and anti-cancer properties of honey [62,63,64,65]. Various enzymes (glucose oxidase, catalase), phenolic acid, flavonoids, ascorbic acids, organic acids, methylglyoxal, and bee defensin-1 are considered to be antibacterial components of honey [63,66].

Manuka honey (MH) is a special type of honey that was used in combination with various scaffolds in in vitro as well as in vivo studies. The reasons for using honey in healing various types of wounds are primarily that MH (as well as other honeys) creates a suitable environment with its low pH, which is conducive to the activity of fibroblasts and macrophages and the production of MGO. Methylglyoxal is considered unique to MH. In addition to these properties, MH interacts with neutrophils, key cells of inflammation, and positively affects wound contraction [67,68,69].

Combinations of MH with various gels (cryogel, gellan gum), mineralized collagen scaffold, and CPs have been studied in vitro and in vivo. The non-cytotoxic effect of MH in combination with tetracalcium phosphate/monetite cements was confirmed in the study by Medvecký et al. [61]. Increased osteoblast proliferation and increased expression of osteoblastic markers such as osteocalcin and osteopontin were also confirmed. According to these authors, the synergistic effect is very suitable and positive for the inclusion of these two-component composites in the in vivo studies.

Collagen scaffolds soaked with 5% and 10% MH were used in an in vivo study by Dewey et al. [44], where they found that 10% manuka concentration was cytotoxic to mesenchymal stem cells. A positive effect on increased osteoprotegerin expression and alkaline phosphatase activity was achieved using a 5% manuka concentration. However, the authors stated that the antibacterial activity of the scaffolds with 5% manuka was not effective against methicillin-resistant *Staphylococcus aureus* (MRSA), which is a very common cause of long-term and complicated hospital-acquired infections. The authors believe that likely, 7% concentration would be possible as an effective inhibitory concentration for MRSA.

Another interesting study that showed an increase in the antibacterial effects of CP cements in combination with manuka is a study by Medvecký et al. [46], where they confirmed and demonstrated the excellent antibacterial effect of MH in α-TCP pastes enriched with glucose oxidase against reference strains of *E. coli* and *S. aureus*. The antibacterial activity of these biocements has increased positively after the addition of MH and glucose oxidase. Since the management of the inflammatory process in implant surgery is crucial for the proper bioacceptability/biocompatibility of implants. Manuka honey without glucose oxidase was also used in our biocements; however, we can state that based on monitoring of animals during the entire postoperative period, there were no local and systemic manifestations of inflammation. Inflammation and its residues were not present even after 6 months after implantation of α-TCP+M25 biocement based on macroscopic, radiological, and histological examination.

Likewise, MH had positive properties on chondrogenesis in vitro, which was investigated using Gellan gum hydrogel in various combinations on human mesenchymal stem cells, where they supported cell proliferation, stimulated the expression of collagen II (key protein of the extracellular matrix of cartilage), the synthesis of glycosaminoglycans and proteoglycans [70,71].

In rat calvarial defects, MH combined with cryogel very effectively stimulated angiogenesis, which is a key factor in all repair processes and determines cell proliferation, differentiation, and overall bone tissue growth. The gel with manuka successfully stimulated bone growth compared to bone char (90% hydroxyapatite) [69].

There are numerous studies evaluating the regenerative potential of CP cements using rat, rabbit, pig, and sheep in vivo models, but only a few evaluate the effect of biocement (especially α-TCP and ß-TCP) on SB.

Bernstein et al. [72] tested the effect of ß-TCP enriched with autologous chondrocytes in a sheep animal model. Based on histology, the authors describe that after 6.5 months, untreated defects were filled with cancellous bone with a thin layer of hyaline cartilage with an irregular surface. On the contrary, in the group treated with ß-TCP, the authors noted the presence of both types of cartilage—hyaline and fibrous. This material also promoted osteoinduction in the early SB, where its original architecture was restored. These findings are consistent with our findings that after the application of the α-TPC+M25 biocement, SB regenerated. The authors also observed, similarly to us, that the regenerative process of the osteochondral defect started from the periphery and continued to the center of the defect.

In addition to the femoral condyles, the *trochlea femoris* is also used as a model for testing various cements in regenerative medicine of osteochondral defects. The effect of ß-TCP+collagen, also enriched with growth factors, was tested in minipigs and compared with unfilled defects for 1.5 months, 3 months, and 1 year [73]. In the control groups, the cartilage had a very similar appearance to our case in the period of 6 months to one year. The cartilage showed signs of fibrillation, had a concave surface, and showed the presence of erosion. Fibrocartilaginous tissue dominated, and the repair of the SB, as in our case, was only partial. The combination of ß-TCP with growth factors had a very good effect, where after a year from the formation of the defect, the cartilage had its original appearance, the same opacity as the original cartilage, and a smooth surface. At the level of the SB, lamellar bone tissue appeared after the therapy, which was completely preserved or present after a year. After half a year, the presence of glycosaminoglycans in the extracellular matrix could be observed. In our case, we chose a follow-up period of 6 months. During six months, the results were similar, and the cartilage did not show signs of degenerative changes after treatment with our biocement. We also observed neocartilage, and in the SB area, mainly woven bone and subsequently lamellar bone tissue were present. These authors also conducted a similar study in 2014 [74], where they studied spontaneous healing of subchondral defects in comparison with ß-TCP+collagen and ß-TCP+collagen+growth factors. Like the previous study, the best results were achieved using ß-TCP with growth factors, where centripetal neobone ingrowth was observed from the sixth week, leading to almost normal bone architecture. Osteoblasts were present in the cementum area, indicating a regeneration process. After 52 weeks of implantation, bone tissue resembling the surrounding bone was present in the defects. The authors also believe that the faster resorption of ß-TCP is achieved precisely by the effect of growth factors that stimulate macrophages and osteoclasts. In our case, it would also be very interesting to test the effect of α-TCP+M25 biocement during a one-year monitoring period. Also, based on our immunohistochemical analyses, remodeling of the subchondral bone defect is still ongoing after six months.

These studies all investigated the effect of ß-TCP on the healing of subchondral defects in animal models. In bone tissue regenerative medicine, α-TCP has been used more and has shown very promising results.

The osteoinductive properties of α-TCP cement with the addition of sodium citrate and alginate were studied by Konishi et al. [42] in a large porcine model (100 kg) on the proximal epiphysis of the tibia for 4 weeks, and they found that after 4 weeks of implantation, the cements were covered with newly formed bone tissue. The bone tissue was formed by osteoblast-like cells. A smooth maturation of the bone tissue was also observed. One disadvantage of this study is the use of a small number of animals and the lack of control groups.

In rats, α-TCP alone or in combination with simvastatin had a similar positive effect. The study confirmed that α-TCP + 0.1 mg simvastatin applied locally to a 5 mm calvarial bone defect in rats supported maximal regeneration of this defect without inflammation. This study nicely demonstrated the positive effect of various bioactive molecules, which correlates with the claims of other authors. In our case, we also assume an improvement in the regenerative capacity of bone tissue and a reduction in the inflammation after the addition of MH [75].

The effect of α-TCP resorption in a porcine model on critical defects of the proximal tibia was studied by Wiltfang et al. [26]. The authors demonstrated that α-TCP is resorbed in comparison with β-TCP. It is also interesting to note that the authors state that the process of resorption of both materials is accompanied by phagocytic activity of large multinucleated cells. An inflammatory reaction was observed in defects where α-TCP was implanted. In our animals, we did not observe the presence of α-TCP+M25 biocement after six months in any case (Figure 15). Centripetal ossification is also observed at the defect site, which is consistent with our observations. Despite the authors’ statement that an inflammatory process was present in their experiment after α-TCP implantation, we did not observe it in our case after 6 months, which may also be associated with the presence of MH as a component of our biocement. After almost 6.5 months, the ceramic materials were partially resorbed and firmly integrated into the surrounding bone; however, almost complete resorption occurred only after 86 weeks, where the authors observed 5% α-TCP residues and 3% β-TCP residues [26].

Current research in the therapy of osteochondral and bone defects is experiencing a large advancement and is trying to develop the most active carrier that will accelerate and support the maximum regenerative capacity of the tissue in order to create the most original tissue. Current trends include the development of various gels and hydrogels [76] with biologically active substances prepared by 3D printers, which can significantly personalize the replaced tissue. The preliminary application of a hydrogel in humans, where a hydrogel–heparin conjugated fibrin was used, is interesting. This hydrogel progressively repaired the cartilage tissue, and based on the MRI examination, there was a good integration of the newly formed cartilage with the native surrounding cartilage [77]. Petcu et al. [78] also mention in their study a very good effect of nanostructured CPs enriched with various biological substances. These materials create suitable scaffolds and mimic the extracellular matrix, creating a place for cell colonization. These characteristics are well known from our previous studies, where our enriched CP materials had better properties than those alone. It is also interesting to note that the pore size also affected cell activity. Hydroxyapatite materials with a pore size of ~400 μm and a porosity of 45.61% have suitable mechanical properties. This size also ensured efficient nutrient transport and cell penetration into the scaffold to form new bone tissue, and incidentally, there is currently a strong trend to use CPs as carriers of various drugs with gradual release [79,80].

In vitro analysis of the biocement itself confirmed its excellent properties; cytotoxicity was not demonstrated, making this material suitable for application. Simple preparation, in the sense of mixing directly in the operating room, makes it a very promising candidate for application in clinical practice. *Post-mortem* analysis demonstrated excellent osteoinductive and osteoconductive properties. Based on radiography, it was possible to monitor its degradation with a minimal amount of residues. Although the control group showed incomplete healing, which we observed macroscopically, the cohesion of the condyle was strong and integrated, despite the fact that on the imaging examination, we observed a radiolucent zone in the defect area. This demonstrates the need to include radiographic methods in the monitoring and evaluation of the regenerative process of osteochondral defects. Histopathological examination correlated with the radiographic findings and confirmed a potentially regenerative effect of α-TPC+M25 biocement.

The promising results of our pilot study highlight the potential of manuka honey-enriched α-tricalcium phosphate biocement in the context of osteochondral defect research. A limitation of our study is that a control group treated only with pure α-TCP biocement was not included. Based on some of our previous studies using pure biocements (especially tetracalcium phosphate/monetit biocement) compared to cements enriched with various bioactive components (amino acids, phytic acid/phytase), we hypothesize the same synergistic effect when using α-TCP with MH [13,28,45]. Our results of the in vivo study were also supported by testing α-TCP and α-TCP+M25 on the osteoblastic line, where cells did not show lower metabolic activity. The pro-osteogenic effect of MH is also based on the study by Medvecký et al. [61], where tetracalcium phosphate/monetite biocement was used as a carrier, where the levels of gene expression of osteogenic markers—collagen type I, osteonectin, osteopontin (after 7 days of cultivation), and osteocalcin (after 14 days of cultivation)—were increased. By day 14, the concentration of osteopontin increased compared to the control without the addition of MH.

## 5. Conclusions

Regeneration of both components, cartilage and subchondral bone, is a key challenge in regenerative medicine of the musculoskeletal system. The potential bioactive effect of calcium phosphate cements with various additives is still being tested and may seem very promising. The new acellular α-tricalcium phosphate biocement enriched with manuka honey showed no cytotoxic properties and was biocompatible. In an in vivo model of osteochondral defect of the medial femoral condyle in pigs, the investigated biomaterial demonstrated positive osteoinductive and osteoconductive properties. Regarding the subchondral bone, the formation of woven and lamellar bone tissue and cartilage regeneration were observed, which was confirmed by Safranin O/Fast Green staining. The osteochondral interface was preserved compared to the untreated defects; the newly formed bone tissue had a typical histoarchitectonics. No biocement residues were observed. The anti-inflammatory effect was demonstrated, and, macroscopically and histologically, we did not detect chronic inflammatory processes or any pathological tissue formation at the level of cartilage and bone tissue in response to the biocement as an exogenous stimulus, even after six months. In the future, the potential use of this two-component biocement in human regenerative medicine could be interesting due to its safety, easy preparation, simple application, and good pre-clinical results on the pig animal model.

## Figures and Tables

**Figure 1:**
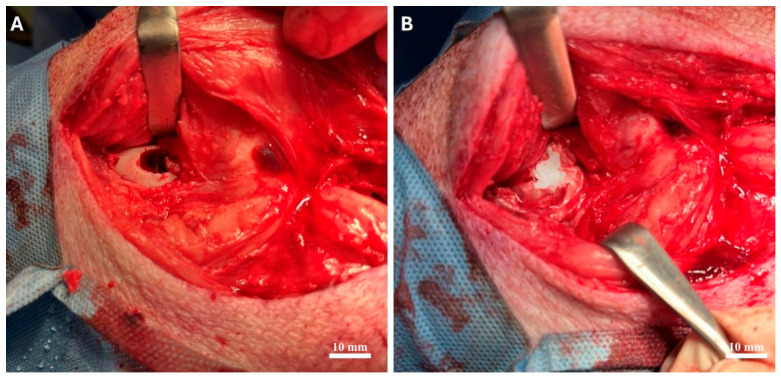
Defect appearance of the left medial femoral condyle before application (**A**) of the biocement and after application (**B**) of the biocement paste.

**Figure 2:**
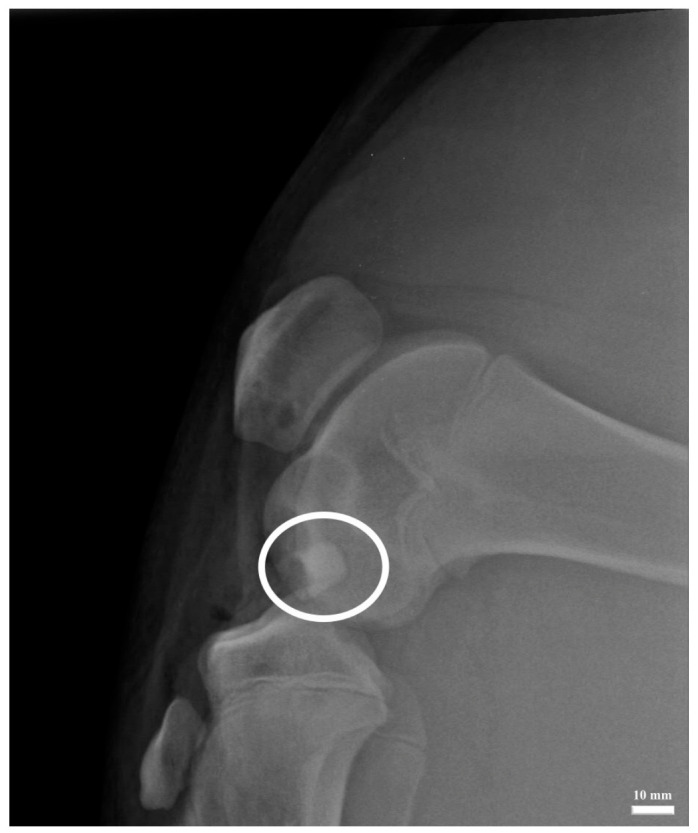
Postoperative radiograph in latero-medial projection immediately after biocement implantation (indicated by circle).

**Figure 3:**
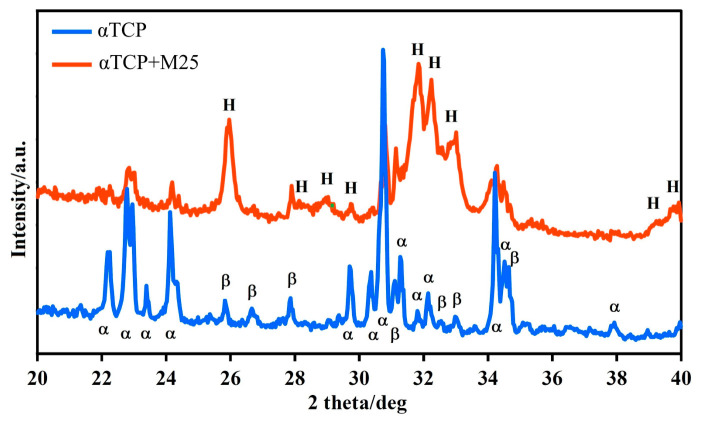
XRD analysis of the origin cement powder (α-TCP) and composite cement (α-TCP+M25) after 7 days of immersion in SBF at 37 °C. (H: hydroxyapatite; α: α-TCP; β: β-TCP).

**Figure 4:**
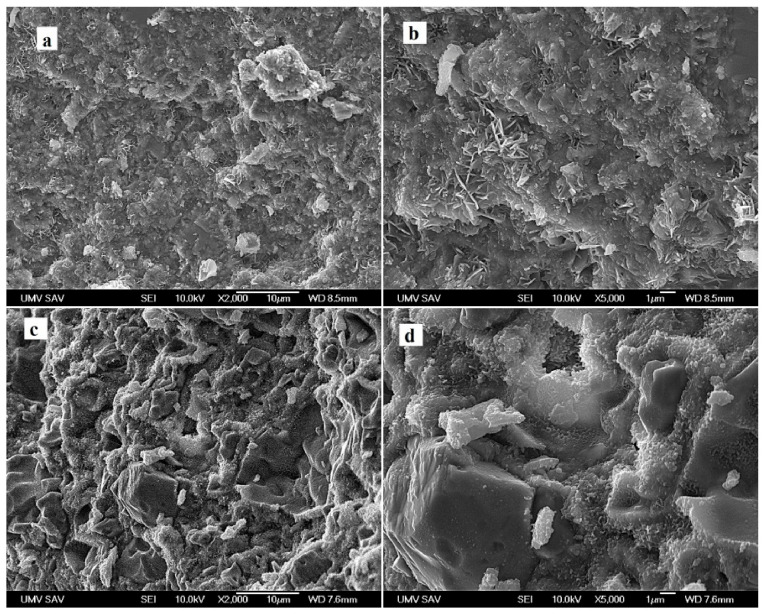
SEM microstructures of α-TCP (**a**,**b**) and α-TCP+M25 (**c**,**d**) cements after 7 days soaking in SBF (Magnification: 2000× (**a**,**c**) and 5000× (**b**,**d**)).

**Figure 5:**
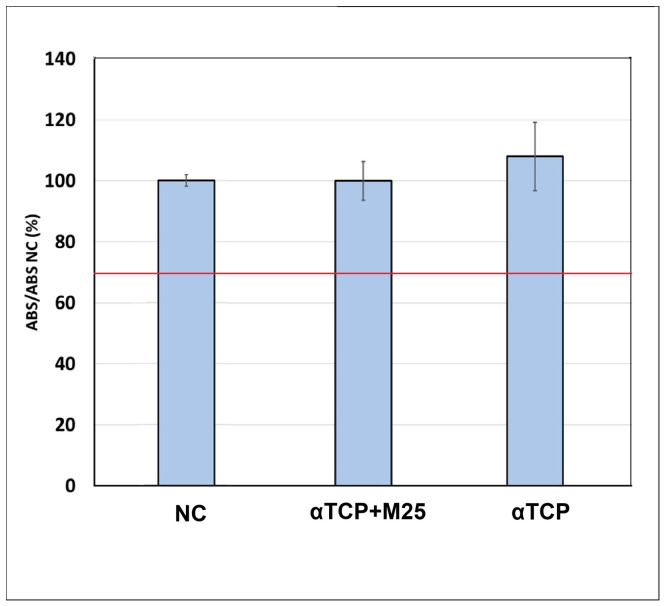
Viability of osteoblasts after 24 h of cultivation in α-TCP and α-TCP + M25 cement extracts. Statistically significant difference (*p* < 0.05) in relation to 70% cytotoxicity threshold (red line).

**Figure 6:**
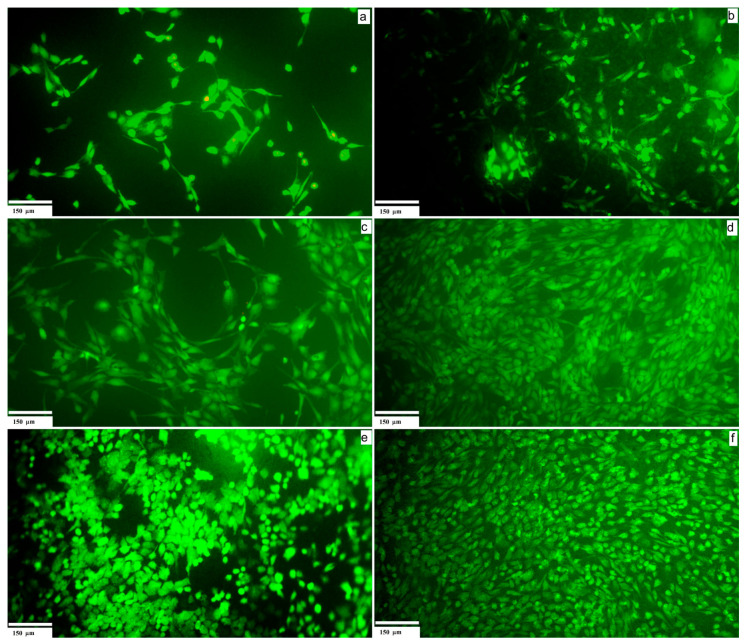
Live/dead fluorescence staining of osteoblasts following 2 days. (**a**): α-TCP; (**b**): α-TCP+M25, 8 days; (**c**): α-TCP; (**d**): α-TCP+M25, 15 days; (**e**): α-TCP; and (**f**): α-TCP+M25 of cultivation on cement surfaces.

**Figure 7:**
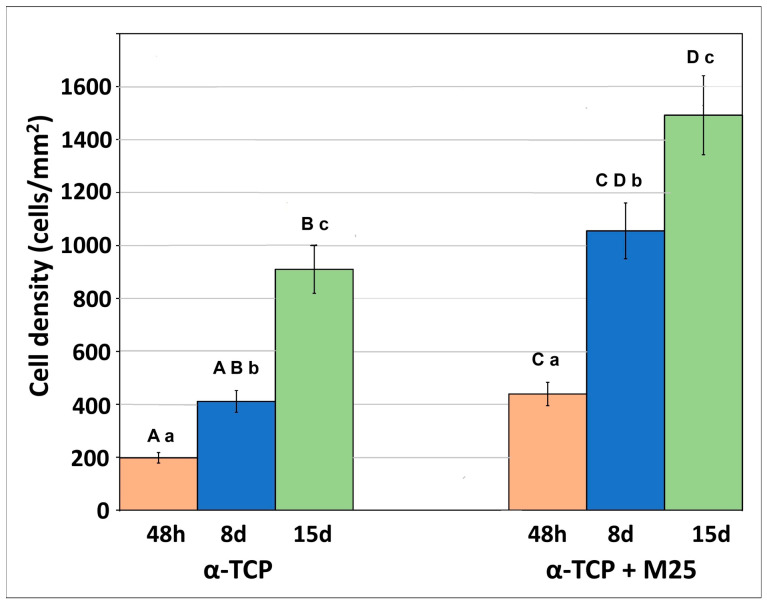
Quantitative evaluation of cell density on α-TCP and α-TCP + M25 surfaces over 15 days. Data are presented as mean ± standard deviation. Statistical significance (*p* < 0.05) was observed for the M25-enriched group compared to the pure α-TCP at all time points. Statistically significant differences between specific groups are indicated by the letters above the bars: uppercase letters above the bars (A, B, C, D) indicate statistically significant differences over time within the α-TCP and α-TCP+M25 group. A: *p* < 0.003; B: *p* < 0.002; C: *p* < 0.001; and D: *p* < 0.004. Lowercase letters (a, b, c) indicate statistically significant differences between the two materials at the same time point: a: *p* < 0.0024; b: *p* < 0.0023; and c: *p* < 0.012.

**Figure 8:**
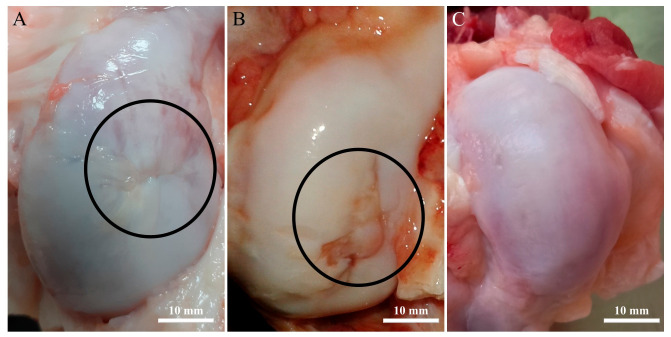
Appearance of newly formed cartilage tissue (circle) after biocement application (**A**); compared to spontaneously healed defect (**B**); and intact cartilage serves as a control for physiological appearance (**C**).

**Figure 9:**
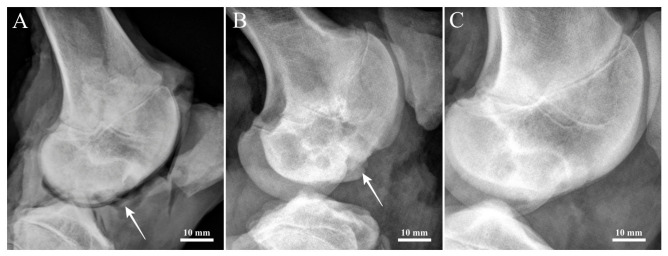
Latero-medial radiograph of the left knee joint 6 months after surgery. Complete healing of the osteochondral defect after application of biocement (**A**) and partial healing of the osteochondral defect without application of biocement (**B**), arrow indicates the defect site. Intact right medial femoral condyle as control (**C**).

**Figure 10:**
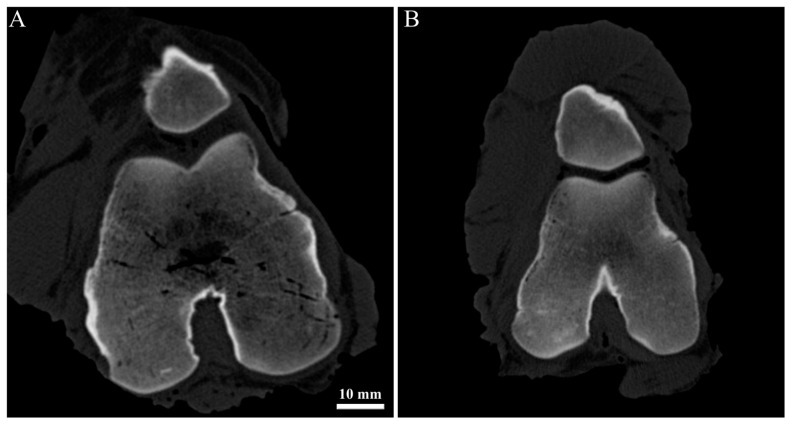
Subchondral and trabecular bone regeneration in an osteochondral defect treated with biocement (**A**), where complete subchondral and trabecular bone regeneration occurred. The degree of regeneration and quality of bone tissue is identical to the control contralateral medial femoral condyle (**B**).

**Figure 11:**
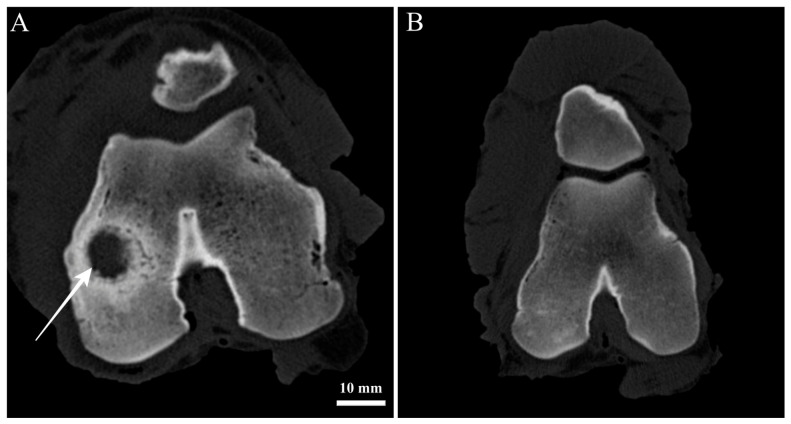
Incomplete and insufficient regeneration of subchondral and trabecular bone in an osteochondral defect healed spontaneously (**A**), where there was no regeneration of subchondral and trabecular bone tissue (arrow) compared to a healthy intact contralateral medial femoral condyle (**B**).

**Figure 12:**
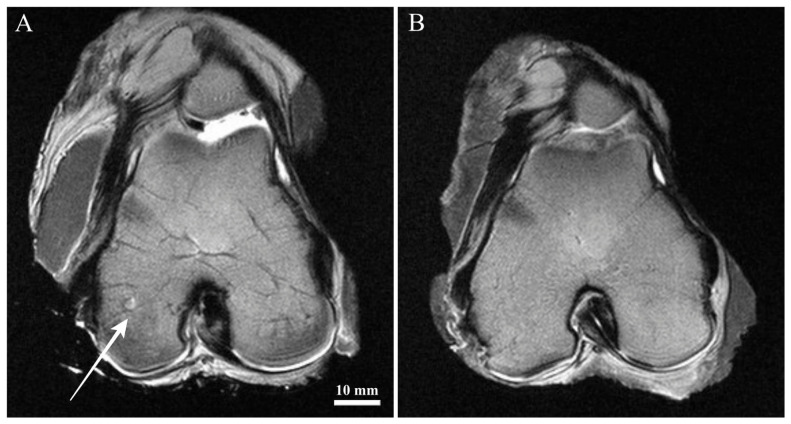
MRI scan indicating the excellent regenerative potential of the biocement. In the operated medial femoral condyle, there was excellent healing of the bone tissue defect with minimal bone changes (arrow) (**A**), which corresponds to the almost identical nature of the intact medial femoral condyle of the contralateral limb (**B**).

**Figure 13:**
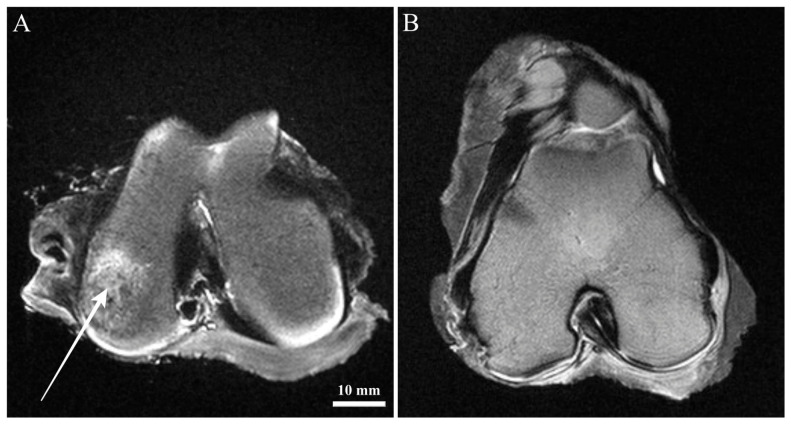
The presence of a partially healed defect of the medial femoral condyle (**A**) after spontaneous healing indicates an irregular organization of the bone tissue (arrow) that does not correspond to the native nature of the medial femoral condyle of the contralateral limb (**B**).

**Figure 14:**
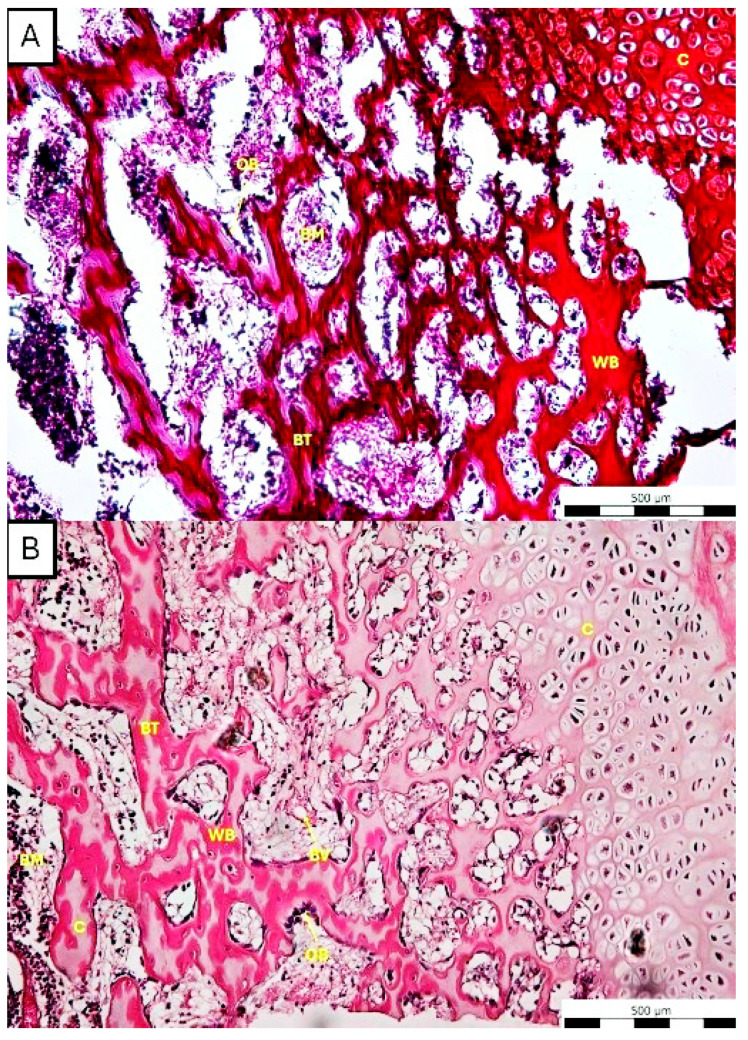
Histological appearance of native articular cartilage (C) and transition to subchondral bone. Representative sections stained with Safranin O/Fast Green (**A**) and hematoxylin–eosin (H&E) (**B**). Safranin O staining demonstrates intact SB containing trabecular bone (BT) and bone marrow (BM) with normal architecture. Normal bone trabeculae with osteoblasts (OB) present on the surface of the bone tissue are visible. Within the bone trabeculae, proteoglycan-rich regions are observed (**A**). The corresponding H&E section confirms the normal morphology of the SB plate, and the underlying trabecular bone (BT) exhibits typical architecture, with bone marrow (BM) occupying the intertrabecular spaces with blood vessels (BV). In some areas of bone tissue, woven bone (WB) is present (**B**).

**Figure 15:**
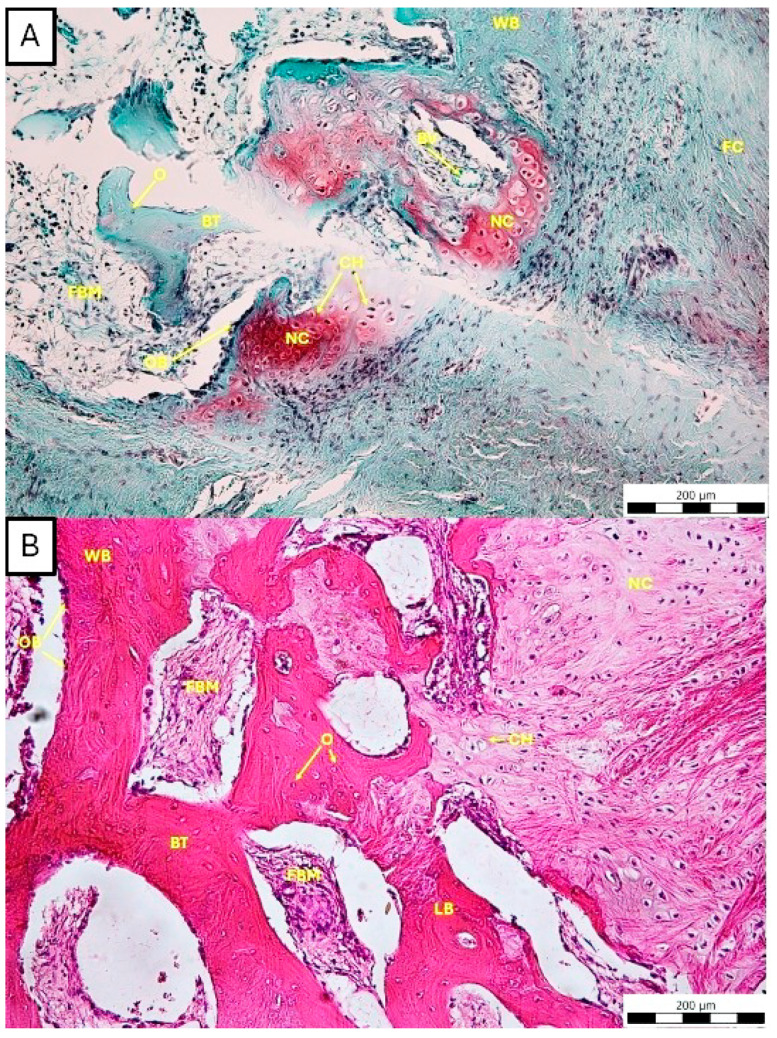
Histological features of osteochondral repair with subchondral bone remodeling. Representative sections stained with Safranin O/Fast Green (**A**) and hematoxylin–eosin (H&E) (**B**). The repair area contains newly formed cartilage (NC) with strong Safranin O positivity, populated by chondrocytes (CH) within lacunae. Adjacent to the cartilaginous regions, bone trabeculae (BT) are present, composed predominantly of woven bone (WB). The trabecular surfaces are lined by osteoblasts (OB), while osteocytes (O) are embedded within the bone matrix. Intertrabecular spaces contain forming bone marrow (FBM) and are penetrated by blood vessels (BV). A surrounding fibrotic callus (FC) is also evident (**A**). The corresponding H&E section shows the transition between new cartilage (NC) and the SB compartment. Bone trabeculae (BT) consist of both woven bone (WB) and areas of more mature lamellar bone (LB). Osteoblasts (OB) line the trabecular surfaces, and osteocytes (O) are present within the bone matrix. Intertrabecular spaces are occupied by forming bone marrow (FBM). The interface between cartilage and bone is well defined, indicating ongoing osteochondral remodeling (**B**).

**Figure 16:**
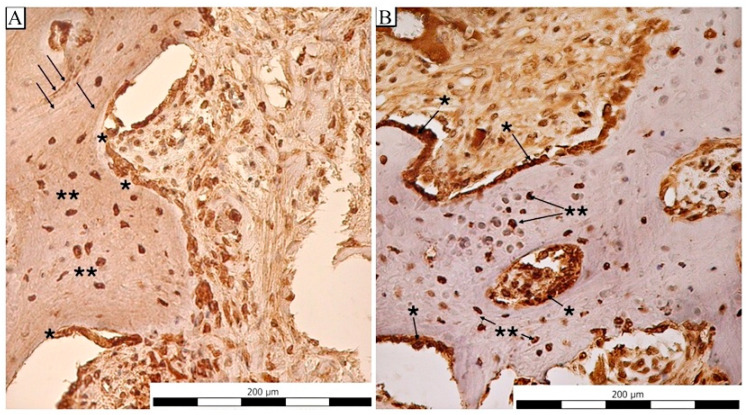
Immunohistochemical staining of the subchondral bone. Immunohistochemical staining for osteocalcin demonstrated strong positivity in osteoblasts (*), with occasional positive osteocytes (**) and a diffuse signal within the extracellular matrix between collagen type I lamellae (arrow), consistent with an active bone formation following the injury (**A**). Immunohistochemical staining for osteopontin demonstrated strong positivity in osteoblasts (*) lining the surface of bone trabeculae of SB, with additional signal detected in a subset of osteocytes (**) and endosteal cells, consistent with active bone remodeling (**B**).

**Figure 17:**
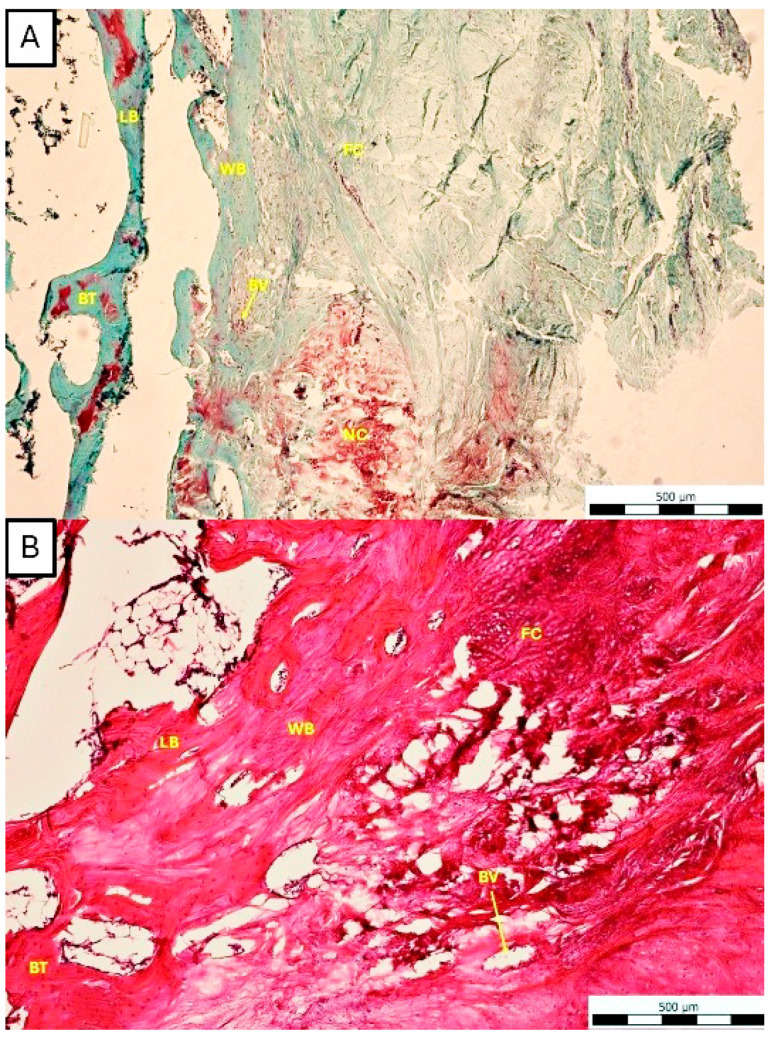
Histological features of subchondral bone during spontaneous healing following a mechanical defect. Representative sections stained with Safranin O/Fast Green (**A**) and hematoxylin–eosin (H&E) (**B**). In the Safranin O/Fast Green section, the subchondral region is visualized with cartilage matrix (NC) highlighted by proteoglycan-associated red staining, while bone and fibrous tissue (FC) appear green, allowing clear distinction between cartilaginous and non-cartilaginous components (**A**). In the corresponding H&E section, overall tissue architecture is emphasized, with bone trabeculae (BT), fibrotic callus (FC), and vascularized (BV) connective tissue more readily identifiable based on cellular and structural morphology rather than matrix composition. Deposits of proteoglycan-rich areas are not visible by H&E staining (**B**). In both sections, the SB region contains irregularly organized trabecular bone (BT) composed predominantly of woven bone (WB) with focal areas of lamellar bone (LB). The defect area is filled with a fibrotic callus (FC) extending into the SB compartment. Intertrabecular spaces contain vascularized connective tissue with blood vessels (BV). At the osteochondral interface, no clearly defined boundary between cartilage and SB is evident.

**Table 1:** Characterization of used antibodies.

Primary and Secondary Antibodies	Antibody Working Dilution	Incubation Parameters	Source/Code
Anti-Osteocalcin primary antibody (OC4-30)	1:200	1 h, 37 °C	Invitrogen (33-5400)
Secondary antibody included in the Ultravision LP detection system, HRP polymer, and DAB plus chromogen	RTU	40 min, RT	THERMO (TL-060-HD)
Anti-Osteopontin primary antibody	1:50	1 h, 37 °C	Invitrogen (PA5-13494)
Secondary antibody included in Ultravision LP detection system, HRP polymer, and DAB plus chromogen	RTU	40 min, RT	THERMO (TL-060-HD)

RT—room temperature, RTU—the antibody supplied by the manufacturer is ready to use.

## Data Availability

Data is contained in this article.
